# Toxicology of caffeine poisoning: molecular mechanisms, toxicokinetic profiles, and clinical implications

**DOI:** 10.3389/ftox.2026.1933375

**Published:** 2026-08-28

**Authors:** Antonia Socias, Rafael Blancas

**Affiliations:** 1 Servicio de Medicina Intensiva, Hospital Universitario Son Llàtzer, Palma de Mallorca, Spain; 2 Servicio de Medicina Intensiva, Hospital Universitario del Tajo, Aranjuez, Spain; 3 Universidad Alfonso X el Sabio, Villanueva de la Cañada, Spain; 4 Fundación para la Investigación Biomédica e Innovación, Hospital Universitario Infanta Sofía y Hospital Universitario del Henares, San Sebastián de los Reyes, Spain

**Keywords:** adenosine receptor antagonism, caffeine poisoning, hemodialysis, hypokalemia, phosphodiesterase inhibition, toxicokinetic

## Abstract

**Background:**

Caffeine toxicity represents a growing public health challenge due to the widespread availability of highly potent formulations. Ingestions of 3–10 g can be fatal, with serious toxicity occurring at plasma concentrations 15 mg/L or greater. This review provides a framework explaining its diverse clinical consequences.

**Methods:**

A comprehensive literature search was conducted across PubMed, Scopus, and Google Scholar using AI-assisted tools, prioritizing clinical, forensic, toxicokinetic, and molecular mechanism studies while excluding chronic moderate consumption.

**Results:**

Caffeine toxicity is dose-dependent, progressing from adenosine receptor antagonism to phosphodiesterase inhibition, intracellular calcium release, and GABA-A antagonism. In overdose, these mechanisms interact synergistically to cause severe neurological, cardiovascular, and metabolic complications. Furthermore, the CYP1A2 metabolic system becomes saturated, prolonging the elimination half-life up to 27 h and causing a disproportionate rise in plasma concentrations. Interactions with drugs like mexiletine drastically reduce clearance.

**Conclusion:**

Severe poisoning stems from complex, synergistic molecular interactions. Hypokalemia serves as a promising, actionable clinical biomarker for severity assessment. When massive ingestions saturate endogenous detoxification, hemodialysis becomes essential for survival. Unregulated markets for pure caffeine require stricter regulatory interventions and intensified clinical surveillance.

## Introduction

Caffeine, or 1,3,7-trimethylxanthine, stands as the most consumed psychoactive compound globally, forming an integral part of the daily routine of millions of people. Today, caffeine is predominantly consumed through popular beverages such as coffee and tea, but it is also a key ingredient in energy drinks and certain pharmaceuticals. A significant contemporary concern is the increasing ease with which pure caffeine, available in powder or tablet form, as well as in highly concentrated energy products, can be acquired through mainstream commerce. This increased accessibility, along with a wider range of caffeine-containing products, has intensified the debate about their safety, potential toxicities, and interactions with other substances.

While moderate consumption of caffeine is generally considered safe, serious toxicities may arise with plasma caffeine concentrations of 15 mg/L or greater (Cappelletti et al., 2018; Maiese et al., 2021; Thelander et al., 2010; Willson, 2018). Concentrations above 50 mg/L are considered toxic, and those above 80 mg/L are considered lethal (Cappelletti et al., 2018; Maiese et al., 2021). Acute ingestion of 3–10 g of caffeine within a short period has been repeatedly associated with fatal outcomes in clinical and forensic case series (Cappelletti et al., 2018; Thelander et al., 2010; Uehlein et al., 2025), although the estimated average fatal oral dose in a healthy adult is considered to be approximately 150–200 mg/kg body weight, equivalent to roughly 10–14 g in a 70-kg adult (Institute of Medicine US Committee on Military Nutrition Research, 2001). This range reflects substantial inter-individual variability in susceptibility rather than a single fixed toxic threshold. Unless otherwise specified, the doses and plasma thresholds discussed in this review refer to healthy adults. A forensic study conducted in Sweden revealed that 1% of 5,000 autopsies had caffeine levels above 10 mg/L, and over a period of 16 years, 20 cases showed levels above 80 mg/L, with arrhythmias being the most common cause of caffeine-related death (Thelander et al., 2010). This evidence underscores the critical need to thoroughly understand the toxicology of caffeine and its clinical implications, given the potential for serious and fatal outcomes.

The convergence of widespread consumption, the increasing availability of highly potent forms that circumvent the self-limiting nature of traditional beverages, and the clear evidence of serious and lethal outcomes, elevates caffeine intoxication from a mere adverse effect to a significant and growing public health concern (Beauchamp et al., 2016; U.S. Food and Drug Administration, 2018). The ease of accidental overdose with the pure forms, in particular, demands urgent attention in terms of public awareness and possible regulatory interventions.

## Methods

### Search strategy and data sources

This narrative review was developed and structured in accordance with the SANRA (Scale for the Quality Assessment of Narrative Review Articles) guidelines to ensure transparency and scientific rigor in the reporting of the evidence presented (Baethge et al., 2019).

A comprehensive literature search was conducted across electronic databases, including PubMed/MEDLINE, Scopus, and Google Scholar, using artificial intelligence (AI) tools to assist in the systematic identification and initial selection of relevant peer-reviewed articles published to date. The search strategy focused on identifying literature related to the mechanisms, clinical manifestations, and management of caffeine poisoning. Search terms included “caffeine poisoning,” “caffeine toxicity,” “energy drinks,” “adenosine receptor antagonism,” “hypokalemia,” and “extracorporeal removal”. Additionally, official reports and safety guidelines from the U.S. Food and Drug Administration (FDA) and the European Food Safety Authority (EFSA) were reviewed to incorporate regulatory perspectives on pure caffeine and energy drink components.

This is a narrative review and not a systematic review; accordingly, a PRISMA flow diagram and an exhaustive count of excluded records were not generated, consistent with the SANRA framework. 210 records; after removing duplicates and screening titles and abstracts against the inclusion/exclusion criteria described above, 52 full-text articles were assessed for eligibility, of which 36 (corresponding to the reference list) were retained for the final narrative synthesis.

### Study selection and inclusion criteria

Articles were considered for inclusion if they provided significant data on the toxicology of caffeine. The selection criteria prioritized:

- Clinical and forensic case reports detailing lethal or severe intoxications.
- Pharmacokinetic and toxicokinetic studies exploring metabolic saturation and half-life alterations.
- Experimental research on the molecular mechanisms of toxicity, such as adenosine receptor antagonism and phosphodiesterase inhibition.

### Exclusion criteria

Studies were excluded if they focused primarily on the chronic effects of moderate caffeine consumption without a toxicological focus, or if they were redundant publications of previously reported cases.

### Data extraction and author consensus

The authors independently screened titles and abstracts for relevance to the review’s objectives. Full-text articles were then evaluated to ensure they addressed the core pillars of the manuscript: molecular mechanisms of toxicity, toxicokinetic profiles, and the resulting clinical implications involving neurological, cardiovascular, and metabolic complications. Disagreements regarding article selection were resolved through discussion and mutual agreement among the authors, ensuring that the final selection provided a comprehensive and updated overview for healthcare professionals.

## Results

**FIGURE 1:**
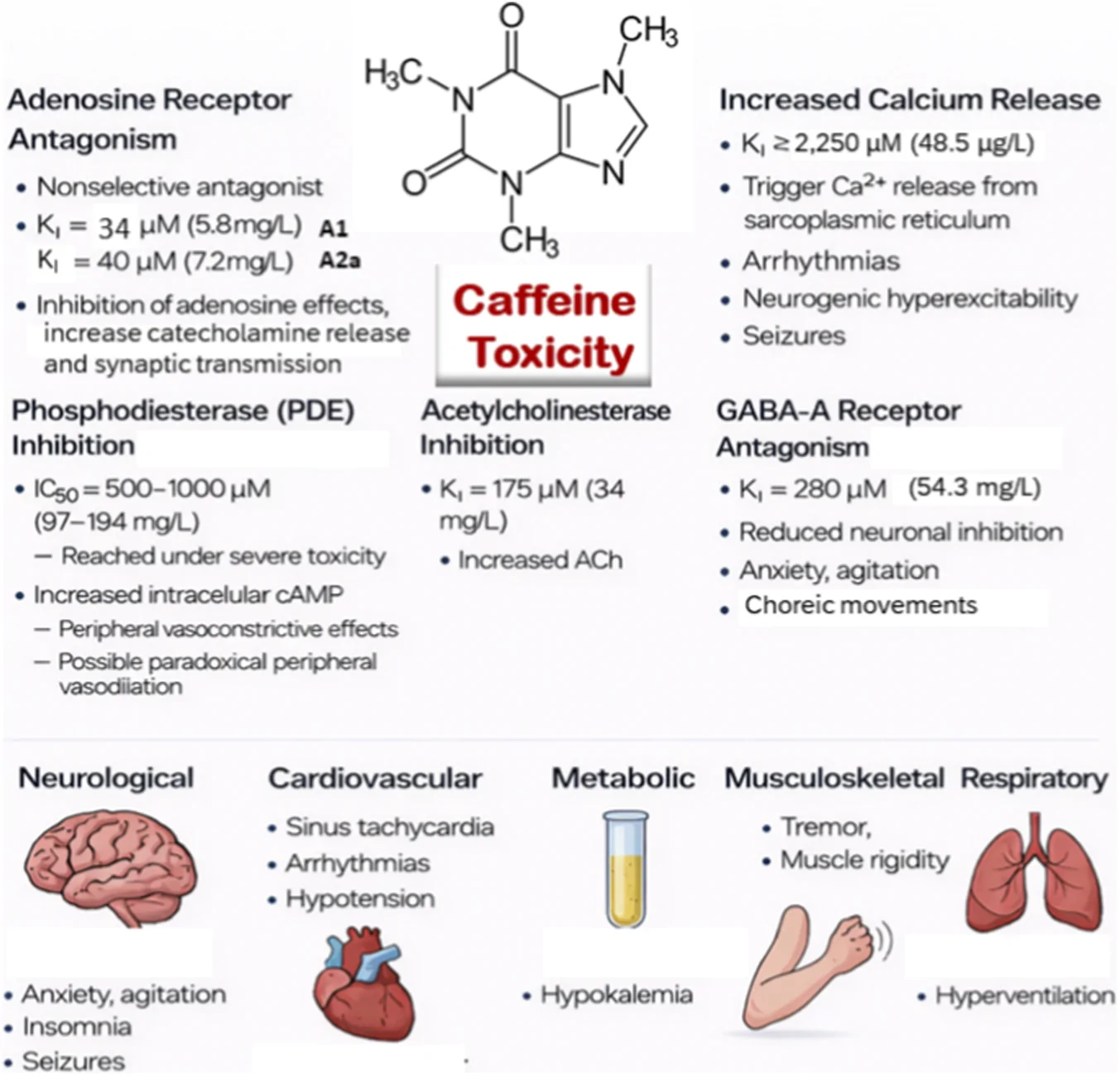
Pharmacodynamic mechanisms and clinical manifestations of caffeine toxicity. Overview of the pharmacological actions and systemic physiological effects of caffeine at toxic levels. The upper panel illustrates molecular mechanisms, including non-selective antagonism of adenosine receptors (A1 and A2a), inhibition of phosphodiesterase (PDE) leading to increased intracellular cAMP, and calcium release from the sarcoplasmic reticulum. At critical concentrations, inhibition of acetylcholinesterase and GABA-A receptor antagonism are observed. T lower section details the resulting clinical manifestations by system: neurological (anxiety, seizures), cardiovascular (tachycardia, arrhythmias), metabolic (hypokalemia), musculoskeletal (tremor, muscle rigidity), and respiratory (hyperventilation). K_I_ and IC_50_ values indicate the concentrations required to trigger these specific effects.

The pharmacological mechanisms of caffeine are intrinsically dependent on the dose consumed, a fundamental principle for understanding the progression of its clinical manifestations, from mild stimulation to severe toxicity (Willson, 2018; Daly et al., 1994). Figure 1 illustrates the main mechanisms of action of caffeine and their associated systemic effects. Caffeine exerts its effects through multiple molecular pathways, which are progressively activated as its concentrations increase in the body. To facilitate clinical interpretation, Table 1 synthesizes the spectrum of clinical toxicity according to plasma caffeine concentration ranges and associated mechanistic thresholds.

**TABLE 1:** Plasma caffeine concentrations and associated clinical manifestations.

Plasma caffeine concentration	Approximate category	Typical clinical manifestations
<10 mg/L	Usual/therapeutic range	Alertness, mild CNS stimulation; range achieved with habitual dietary consumption
10–15 mg/L	Mild toxicity	Nervousness, anxiety, insomnia, tremor, mild sinus tachycardia, gastrointestinal upset
15–50 mg/L	Moderate–severe toxicity	Marked tachycardia, hypertension, agitation, vomiting, hypokalemia, tremor/muscle rigidity, hyperventilation
50–80 mg/L	Severe toxicity	Supraventricular/ventricular arrhythmias, hypotension, seizures, rhabdomyolysis, metabolic (lactic) acidosis
>80 mg/L	Potentially lethal	Refractory ventricular arrhythmias, cardiovascular collapse, multiorgan failure, death

Data adapted and synthesized from Willson (2018) (Willson, 2018) and Cappelletti et al. (2018) (Cappelletti et al., 2018). Therapeutic plasma concentrations (<10 mg/L) represent range achieved with typical dietary intake (200–400 mg). Mild-to-moderate toxic effects arise from competitive inhibition of adenosine A1 and A2a receptors. Severe toxicity (>50 mg/L) and potentially lethal outcomes (>80 mg/L) involve phosphodiesterase inhibition, increased intracellular cAMP, and massive release of endogenous catecholamines, often complicated by CYP1A2 metabolic saturation.

### Adenosine receptor antagonism

The main pharmacological effect of caffeine is its action as a non-selective antagonist of adenosine receptors (A1, A2a, A2b and A3). As shown in Figure 1, the inhibition constants (KI) are 34 μM (6.8 mg/L) for A1 receptors and 40 μM (7.2 mg/L) for A2a receptors. This antagonism disrupts the normal physiological effects of adenosine, which include sleep regulation, cardiovascular function, and neurotransmission. The direct consequence of this inhibition is an increase in catecholamine release and an alteration of synaptic transmission, contributing to the stimulant effects of caffeine (Willson, 2018; Ribeiro and Sebastião, 2010; Górska and Gołembiowska, 2015).

### Phosphodiesterase (PDE) inhibition

At higher concentrations, typically achieved in cases of severe toxicity, caffeine inhibits the enzyme phosphodiesterase (PDE). The IC50 for this effect is between 500–1,000 μM (97–194 mg/L), levels that are associated with severe toxicity. This inhibition results in an increase in intracellular cyclic adenosine monophosphate (cAMP). The implications of this increase in cAMP include peripheral vasodilation, which paradoxically can lead to hypotension in situations of severe overdose (Willson, 2018).

### Increased intracellular calcium release

At very high concentrations, specifically above 2,250 μM (equivalent to 48.5 mg/L), caffeine triggers the release of calcium from the intracellular deposits of the sarcoplasmic reticulum. This increase in intracellular calcium contributes significantly to the occurrence of cardiac arrhythmias, neuromuscular hyperexcitability and seizures (Porta et al., 2011; Okudaira et al., 2014).

### Inhibition of potassium channels (hERG)

Hypokalemia, frequently observed during caffeine overdose, is thought to result primarily from stimulation of the Na+/K+-ATPase pump (Magkos and Kavouras, 2005). Although this electrolyte disturbance is commonly attributed to β2-adrenergic activation secondary to catecholamine release, additional mechanisms may contribute, including adenosine receptor antagonism and phosphodiesterase inhibition, particularly in severe intoxication (Willson, 2018; Reid et al., 1986). At very high concentrations, caffeine blocks hERG in an open state. That can cause long QT syndrome and arrhythmias (Willson, 2018; Zheng et al., 2017).

Rapid correction of hypokalemia is paramount. Potassium supplementation (e.g., 10 mEq/hour intravenously) is given, particularly in patients with symptomatic hypokalemia, especially in the presence of muscle weakness, ventricular arrhythmias, abnormal T waves, or prolonged QTc intervals (Tsuji et al., 2020).

### GABA-A receptor antagonism

At toxic concentrations (KI = 280 μM or 54.3 mg/L), caffeine acts as an antagonist of GABA-A receptors, resulting in decreased neuronal inhibition (Willson, 2018; Ribeiro and Sebastião, 2010; Kardos and Blandl, 1994). This mechanism can directly contribute to the occurrence of seizures, agitation, and cholinergic overactivity, exacerbating the neurological effects of intoxication.

### Acetylcholinesterase inhibition

Also, at toxic concentrations (KI = 175 μM or 34 mg/L), caffeine can inhibit the enzyme acetylcholinesterase. This inhibition results in increased availability of acetylcholine (ACh) in synapses, which may contribute to the cholinergic overactivity seen in cases of severe poisoning (Pohanka and Dobes, 2013).

Caffeine exhibits multi-target pharmacology, meaning that the clinical presentation of severe intoxication is not due to a single pathway, but to a complex synergistic interaction of several mechanisms. This inherent complexity makes clinical diagnosis difficult and calls for a comprehensive and supportive treatment approach that addresses the various symptomatic pathways, rather than relying on a single specific antidote. Understanding this overlapping mechanistic landscape is critical to anticipating and managing the full spectrum of potential clinical manifestations.

### Pharmacokinetics and toxicokinetics of caffeine: absorption, distribution, metabolism and elimination

Caffeine is rapidly and almost completely absorbed in the gastrointestinal tract, reaching peak plasma concentrations 30–120 min after oral ingestion (Newton et al., 1981). Once absorbed, it is widely distributed throughout the body, easily crossing the blood-brain barrier and the placenta (Chen et al., 2010). Its average volume of distribution is 0.7 L/kg. Protein binding is low (10%–35%) (Holstege et al., 2003). Caffeine metabolism is primarily hepatic, largely mediated by the cytochrome P450 1A2 (CYP1A2) enzyme system. This enzyme is responsible for converting caffeine into three main metabolites: paraxanthine (84%), theobromine (12%), and theophylline (4%) (Chen et al., 2010; Carrillo and Benitez, 2000). Importantly, paraxanthine, the most abundant metabolite, is itself psychoactive and contributes to the overall effects of caffeine (Willson, 2018).

The elimination half-life of caffeine in healthy adults ranges from 2.5 to 10 h (Pohanka and Dobes, 2013), but can vary significantly due to a variety of factors, including genetic polymorphisms of CYP1A2, the presence of liver disease, pregnancy, and drug interactions (Kardos and Blandl, 1994). People who slowly metabolize caffeine due to variations in CYP1A2 may experience prolonged effects and increased susceptibility to adverse events.

In terms of ingested dose relative to body weight, pediatric ingestions exceeding approximately 30 mg/kg are generally considered to warrant clinical evaluation, whereas the estimated fatal adult dose corresponds to approximately 150–200 mg/kg (Institute of Medicine US Committee on Military Nutrition Research, 2001).

### Pharmacokinetic alterations in overdose

High doses of caffeine have the potential to saturate metabolic pathways, leading to a prolongation of the elimination half-life and maintenance of high plasma concentrations. A documented case illustrates this phenomenon: a 25-year-old Japanese woman who intentionally ingested 5.9 g of caffeine had an apparent terminal elimination half-life of 27 h during the first 2 days of hospitalization, a substantially longer duration than the typical half-life. In this case, plasma concentrations of caffeine and its main metabolite, paraxanthine, remained significantly elevated for prolonged periods, highlighting the difficulty in managing these poisonings (Carrillo and Benitez, 2000; Adachi et al., 2021).

Metabolic saturation means that the body’s ability to process and eliminate the drug is overwhelmed, leading to a disproportionate increase in the concentration of the drug and a significantly extended duration of its action, even after initial ingestion. Clinically, this directly dictates the need for prolonged observation of the patient, the limited efficacy of activated charcoal in single doses if absorption has already occurred, and the need to consider aggressive extracorporeal elimination methods (such as hemodialysis) due to insufficient natural detoxification mechanisms (Holstege et al., 2003). These prolonged half-lives require continuous monitoring and often intensified elimination strategies in cases of severe poisoning.

### Drug interactions with drugs

Caffeine’s interactions with various medications stem primarily from its influence on the activity of the CYP1A2 enzyme. Drugs that inhibit CYP1A2, such as fluoroquinolones, fluvoxamine, and mexiletine, can significantly reduce caffeine elimination, resulting in elevated caffeine levels and an increased risk of toxicity. Conversely, drugs that induce its activity, such as carbamazepine and phenobarbital, can speed up caffeine metabolism (Kardos and Blandl, 1994; Carrillo and Benitez, 2000).

A particularly noticeable and serious interaction may occur with mexiletine, a class 1B antiarrhythmic drug. Co-ingestion of high doses of caffeine and mexiletine can lead to profound toxicity (Joeres et al., 1987; Wang et al., 2004). The case of a 20-year-old woman who ingested 18 g of caffeine and 3500 mg of mexiletine has been documented, resulting in severe intoxication characterized by hypokalemia, lactic acidosis, and hemodynamic instability. The clearance of caffeine calculated from blood concentrations between 35.5 h and 59.5 h after arrival, when hemodialysis (HD) was not performed, was 25.3 mL/min, which was significantly reduced compared to the estimated clearance range of 42–126 mL/min for a physically healthy adult. If HD had not been performed, it might have taken longer to wean off VA-ECMO due to reduced caffeine clearance in the presence of mexiletine (Kohara et al., 2024).

### Interactions with drugs of abuse and new psychoactive substances (“legal highs”)

There is growing concern about the simultaneous intake of energy drinks and caffeine with illicit drugs and New Psychoactive Substances (NPS), often referred to as ‘legal highs’. These substances are marketed as legal alternatives to traditional illicit drugs and may contain undeclared or variable amounts of psychoactive compounds, including high concentrations of caffeine.

Studies have revealed that many products sold as “legal highs” contain caffeine as their main or only active pharmacological ingredient, sometimes in very high percentages (up to 96%) (Davies et al., 2012). The stimulant effects of caffeine can be perceived as similar to those of weaker recreational drugs, making it an attractive, albeit dangerous, component in these unregulated products. The combination of caffeine with other stimulants present in NPS or drugs of abuse can cause exaggerated sympathomimetic effects, increasing the risk of cardiotoxicity, seizures and serious psychological disorders (Gurley et al., 2015). Patients presenting with acute recreational drug toxicity should be evaluated for possible concomitant intake of high-dose caffeine, as this can significantly influence clinical presentation and management strategies. The lack of clear labelling and quality control in the market for “legal highs” poses a substantial risk to public health due to unpredictable doses of caffeine and potential interactions with other undisclosed psychoactive compounds.

### Additional components of energy drinks (taurine, inositol, glucuronolactone)

Beyond their high caffeine content, energy drinks often contain other stimulants such as guarana (a significant source of caffeine), taurine, B vitamins, and sugar. The synergistic effects of these various ingredients, combined with caffeine, can amplify their stimulant properties and increase the risk of adverse interactions. Some energy drinks can contain up to 250 mg of caffeine per container (Wolk et al., 2012).

Taurine is an amino acid present in the CNS, striated muscle, and lungs. It is known to increase excitatory potentials in synapses and axons by acting on low-voltage calcium channels, similar to tetanus stimulation (Chepkova et al., 2002). It shortens the reaction time to stimuli, enhances the feeling of wellbeing and improves sociability, effects that are enhanced by alcohol. Although it interacts with inositol and caffeine, the European Food Safety Authority (EFSA) has concluded that the levels of taurine used in energy drinks do not pose a safety concern (Aguilar et al., 2009; Seidl et al., 2000).

Inositol, a component of the vitamin B complex and part of the cell membrane phospholipids, is involved in the regulation of cell membrane responses, thus enhancing the effects of taurine and caffeine. Inositol can also induce a state of mania independently (Willson, 2018).

Glucuronolactone (D-glucurone-γ-lactone) is a naturally occurring substance in the human body that is a structural part of connective tissues and is involved in detoxification processes (Tamura et al., 1968). EFSA determined in 2009 that exposure to glucuronolactone from regular consumption of energy drinks is not a safety concern, with a no-observed-adverse-effect level of 1,000 mg/kg/day. EFSA also considers glucuronolactone unlikely to interact with caffeine, taurine, alcohol or the effects of exercise (Aguilar et al., 2009).

## Discussion

The findings of this review show that severe caffeine poisoning cannot be explained by a single mechanism, but rather by the combined effect of several pathways that become progressively engaged as plasma concentrations rise. At high doses, caffeine is no longer simply a stimulant; it affects multiple systems at once, which helps explain why the clinical picture can be so variable and, at times, difficult to manage. In practice, this means we are not dealing with a simple toxidrome, but with a complex poisoning in which neurological, cardiovascular, and metabolic disturbances overlap. This mechanistic synergy is reinforced by both recent original clinical analyses (Uehlein et al., 2025; Han et al., 2022) and experimental animal models demonstrating caffeine-induced arrhythmogenesis through aberrant calcium dynamics (Okudaira et al., 2014; Hamad, 2024).

The toxicokinetic parameters and clinical thresholds described above pertain principally to healthy, non-pregnant adults; important differences exist in vulnerable populations. During pregnancy, CYP1A2 activity progressively declines, prolonging the caffeine half-life up to approximately 15 h by the third trimester, with implications for maternal accumulation and fetal exposure (Murray and Traylor, 2026; National Institute of Child Health and Human Development, 2026). Neonates and young infants have markedly immature CYP1A2 activity, with half-lives of up to 8 h in full-term infants and approximately 100 h in preterm infants (National Institute of Child Health and Human Development, 2026; Evans et al., 2024), while young children are also disproportionately vulnerable to accidental toxicity at comparatively low absolute doses because of their lower body weight, as recently illustrated by a pediatric case of caffeine-induced rhabdomyolysis and seizure-like activity (Stephanos and Hayes, 2025). In older adults, reduced hepatic reserve, polypharmacy, and a higher prevalence of CYP1A2-inhibiting medications may prolong caffeine clearance and increase susceptibility to cardiovascular complications (Murray and Traylor, 2026), although dedicated toxicokinetic data in this population remain scarce. These considerations argue for individualized risk assessment rather than reliance on fixed adult thresholds.

From a clinical perspective, one of the most useful observations from this review is the consistent association between caffeine overdose and hypokalaemia. Although it is not a specific marker, serum potassium may offer a rapid and accessible clue to the severity of intoxication, particularly when direct caffeine measurement is not immediately available. Another important point is the prolongation of the half-life in massive overdoses: once metabolism becomes saturated, the clinical course no longer follows the usual pattern, and patients may remain with elevated concentrations for much longer than expected. This requires prolonged observation and means the duration of risk should not be underestimated.

The experience with pharmacological interactions, such as the one described with mexiletine, also reminds us that caffeine poisoning should rarely be interpreted in isolation. For that reason, a careful medication history remains one of the most valuable tools in these cases.

In severe or refractory presentations, haemodialysis should be considered early, not merely as a rescue measure, but as a reasonable part of management when endogenous elimination is insufficient (Han et al., 2022).

The availability of pure caffeine in powder or tablet form removes the sensory barrier that exists with ordinary beverages and makes accidental massive ingestion more likely. Added to this is the presence of caffeine in unregulated products, energy drinks, and mixtures with other stimulants, which increases uncertainty about the actual dose and the risk of combined toxicity. In this sense, the issue is not only pharmacological, but also regulatory and educational.

## Limitations

This study has several limitations that should be kept in mind. As a narrative review, the selection of the literature may still be affected by a degree of bias, even when guided by a framework such as SANRA. In addition, much of the available evidence comes from isolated case reports or small series, which limits the strength of the conclusions. It is also true that the use of AI-assisted tools in the literature search may have improved efficiency, but it does not completely remove the possibility of selection bias. For these reasons, some of the associations described here should be interpreted with caution.

## Future research directions

Looking ahead, prospective multicentre studies would be useful to validate severity markers such as hypokalaemia and to define more clearly when extracorporeal elimination should be initiated. Further pharmacokinetic studies are also needed to better understand how caffeine metabolism becomes saturated in overdose and how factors such as age, pregnancy, liver disease, and genetic variability influence this process. In parallel, stricter surveillance of the actual caffeine content in energy drinks and unregulated products would help quantify the real-world burden and inform more effective preventive measures.

## Conclusion

Caffeine remains a ubiquitous compound in modern society, offering both perceived benefits and significant health risks, particularly in overdose scenarios and among vulnerable populations. The ease of access to highly concentrated caffeine products and the burgeoning energy drink market have introduced new dimensions to its clinical toxicology. For healthcare professionals, it is paramount to understand the dose-dependent mechanisms of action, which range from antagonism of adenosine receptors at low concentrations, to inhibition of phosphodiesterase, increased intracellular calcium release, antagonism of GABA-A receptors, and inhibition of acetylcholinesterase at toxic concentrations. The severe clinical presentation of caffeine poisoning is not the result of a single pathway but rather a complex synergistic interaction of these multiple molecular mechanisms.

It is also crucial to understand the intricate pharmacokinetics influenced by genetic and environmental factors, as well as the complex interactions with other substances, including medications (such as mexiletine) and new psychoactive substances. In overdose situations, the CYP1A2 metabolic system becomes saturated, shifting kinetics and significantly prolonging the elimination half-life—documented up to 27 hours—which leads to a disproportionate increase in plasma concentrations.

In this context, hypokalemia has emerged as a promising and actionable clinical biomarker for the rapid assessment of toxicity severity. Although supportive care remains the cornerstone of treatment, advanced interventions such as hemodialysis are critical and essential in severe cases where endogenous metabolic pathways are overwhelmed by saturation.

Future research should focus on optimizing therapeutic strategies for severe poisoning, identifying new biomarkers for early detection, and elucidating the long-term health consequences of chronic high-dose caffeine consumption.
